# Cellular aging dynamics after acute malaria infection: A 12‐month longitudinal study

**DOI:** 10.1111/acel.12702

**Published:** 2017-11-16

**Authors:** Muhammad Asghar, Victor Yman, Manijeh Vafa Homann, Klara Sondén, Ulf Hammar, Dennis Hasselquist, Anna Färnert

**Affiliations:** ^1^ Unit of Infectious Diseases Department of Medicine Solna Karolinska Institutet Stockholm Sweden; ^2^ Department of Infectious Diseases Karolinska University Hospital Stockholm Sweden; ^3^ Unit of Biostatistics Department of Epidemiology Institute for Environmental Medicine Karolinska Institutet Stockholm Sweden; ^4^ Department of Biology Lund University Lund Sweden

**Keywords:** CDKN2A, cellular aging, Malaria, *Plasmodium falciparum*, Telomerase, Telomeres

## Abstract

Accelerated cellular aging and reduced lifespan have recently been shown in birds chronically infected with malaria parasites. Whether malaria infection also affects cellular aging in humans has not been reported. Here, we assessed the effect of a single acute *Plasmodium falciparum* malaria infection on cellular aging dynamics in travelers prospectively followed over one year in Sweden. DNA and RNA were extracted from venous blood collected at the time of admission and repeatedly up to one year. Telomere length was measured using real‐time quantitative PCR, while telomerase activity and CDKN2A expression were measured by reverse transcriptase (RT)–qPCR. Our results show that acute malaria infection affects cellular aging as reflected by elevated levels of CDKN2A expression, lower telomerase activity, and substantial telomere shortening during the first three months postinfection. After that CDKN2A expression declined, telomerase activity increased and telomere length was gradually restored over one year, reflecting that cellular aging was reversed. These findings demonstrate that malaria infection affects cellular aging and the underlying cellular mechanism by which pathogens can affect host cellular aging and longevity need to be elucidated. Our results urge the need to investigate whether repeated malaria infections have more pronounced and long‐lasting effects on cellular aging and lifespan (similarly to what was observed in birds) in populations living in malaria endemic areas.

## 1 INTRODUCTION

Increasing chronological age typically results in a loss of physical capability and increased age‐related morbidities. Aging may be the cause of diseases, but infectious diseases can also act as exogenous drivers of aging and the underlying mechanisms may include enhanced inflammation, pathogen‐dependent tissue destruction, or accelerated cellular aging through increased cell turnover (Asghar, Hasselquist, et al., 2015; Asghar et al., 2016; Gavazzi & Krause, 2002). Recently, we have shown that chronic asymptomatic malaria infections resulted in reduced lifespan, mediated through faster telomere shortening in birds (Asghar, Hasselquist, et al., 2015; Asghar et al., 2016).

Telomeres, the end parts of chromosomes that consist of tandem repeats of noncoding DNA, shorten as part of normal aging (Blackburn, Epel, & Lin, 2015) and also due to various types of intrinsic and extrinsic factors (Blackburn & Epel, 2012; Epel et al., 2004). Shorter telomere length has been linked to human pathologies such as cardiovascular disease, stroke, diabetes, dementia, and immune dysfunction (Atzmon et al., 2010). Large cohort studies have shown that shorter telomere length is a significant predictor of all‐cause mortality even after controlling for age and other known risk factors (Blackburn et al., 2015; Needham et al., 2015; Rode, Nordestgaard, & Bojesen, 2015). To counteract the progressive telomere shortening, stem cells and progenitor cells as well as a majority of cancer cells express telomerase (an enzyme capable of adding the telomeric repeats); however, in somatic cells, telomerase is downregulated to avoid tumor formation (Artandi & DePinho, 2010). Over the last decade, cyclin‐dependent kinase inhibitor 2A (CDKN2A*)*, a cell cycle inhibitor, has emerged as a valuable candidate biomarker of cellular aging (Jeck, Siebold, & Sharpless, 2012; Shiels, 2010). The expression of CDKN2A is highly suppressed during early development (Li et al., 2009) and remains very low in young mammals (Burd et al., 2013). CDKN2A is progressively expressed in peripheral blood and in most tissues, and its expression level correlates with age in mice and human (Sorrentino, Sanoff, & Sharpless, 2014). Furthermore, CDKN2A expression in tissues correlates negatively with organ function (Koppelstaetter et al., 2008; Sorrentino et al., 2014).

About half of the global human population lives in malaria endemic areas and ~200 million cases of malaria with ~400,000 deaths were reported in 2015 (WHO 2016). Efficacious treatment of uncomplicated *Plasmodium falciparum* malaria leads to an apparent complete recovery. However, the risk of any long‐term adverse effect is poorly understood. Recent studies showed that malaria infection in birds results in accelerated telomere shortening in peripheral blood and other tissues (liver, lungs, kidney, heart, spleen, and brain), and persistent chronic asymptomatic malaria infections result in reduced lifespan, implying that a seemingly mild disease accelerates cellular aging (Asghar, Hasselquist, et al., 2015; Asghar et al., 2016; Karell, Bensch, Ahola, & Asghar, 2017). Furthermore, induced *Salmonella* infection in mice (Ilmonen, Kotrschal, & Penn, 2008) leads to accelerated telomere shortening in blood. Nonetheless, the effect of malaria, or other acute infections, on cellular aging has not been reported in humans.

It is difficult to establish a causal relationship between disease and cellular aging in humans. However, studies of acute infections that are cured after treatment, that is, reflecting a temporary event, can be used as a model to understand the effect of diseases on cellular aging, and thus have the potential to establish causality. Here, we investigated whether a single acute malaria infection affects cellular aging by examining the dynamics and interplay of telomere length, telomerase activity, and CDKN2A expression in travelers successfully treated for *P. falciparum* malaria at Karolinska University Hospital in Stockholm, Sweden. The study design includes individuals that are followed prospectively in a malaria‐free setting over one year, thus without risk of re‐exposure, offering a unique opportunity to study both short‐ and long‐term effects of single malaria episodes on cellular aging dynamics.

## 2 RESULTS

### 2.1 Telomere length dynamics

**Figure 1:**
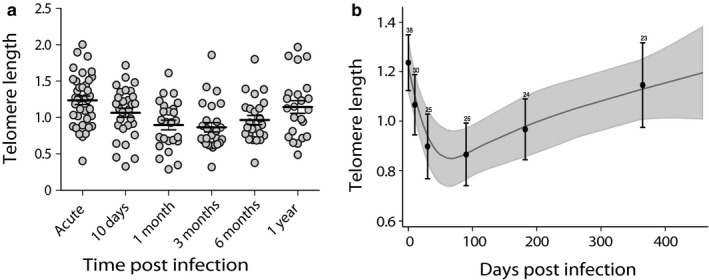
Telomere length dynamics in blood of travelers followed over one year after successful treatment of *Plasmodium falciparum* malaria (*n *= 38). (a) Telomere length dynamics after single acute malaria infection in peripheral blood; each gray circle represents the telomere measurement of individual sample, solid black lines represent the mean at each sampling point, and error bars denote the 95% CI. (b) Telomere length, as predicted from the mixed models; solid gray line denotes the mean telomere length and shaded gray areas denote the 95% CI of the model predictions when adjusted for covariates. Black dots denote the observed mean telomere length at each time point (pooled data at approximate measurement day), and error bars denote the 95% CI of the mean. Number on each error bar represents the sample size at each measurement time point

Telomere length in peripheral blood cells was significantly reduced between day 0 and day 10 postinfection (paired t test mean diff = −0.14, 95% confidence interval of mean difference (CI) −0.02 to −0.25, *N *= 30, *p *= .022) (Figure 1a), and the shortest telomere length was observed at 3 months postinfection compared to day 0 (paired t test mean diff = −0.34, 95% CI −0.19 to −0.48, *N *= 26, *p *< .001) (Figure 1a). Telomere length at 12 months postinfection was significantly longer compared to 3 months postinfection (mean diff = 0.39, 95% CI 0.58 to 0.21, *N *= 17, *p *< .001) and there was no significant difference in telomere length at 12 months postinfection compared to day 0 (mean diff = −0.07, 95% CI 0.12 to −0.26, *N *= 23, *p *= .460, Table S1) (Figures 1a and S1). The mixed model showed that telomere length significantly changed over the one‐year follow‐up (Wald χ^2^ = 36.50, *N *= 166, *p *< .001) (Figure 1b) with no significant effect of age, sex, number of febrile days before treatment, parasite density, disease severity and patient origin (all *p *≥ .372, Table 1). Furthermore, telomere length dynamics were not affected by type of antimalarial treatment (*p *= .515).

**Table 1:** Association between host factors and telomere length dynamics in blood of travelers over one‐year follow‐up after a single successfully treated *Plasmodium falciparum* malaria infection (*N *= 38, observatio*n *= 166)

Factors	Unit	Univariate analysis			Multivariate analysis		
		Coef.	SE	*p*‐value	Coef.	SE	*p*‐value
Male	Yes	−0.08	0.09	.400	−0.02	0.12	.852
Age	Years	−0.001	0.004	.715	−0.004	0.004	.852
Febrile days	Days	0.003	0.02	.860	0.005	0.02	.779
Parasite intensity	Parasites/ml	−0.13	0.23	.579	−0.001	0.03	.993
Severe malaria^a^	Yes	−0.26	0.10	.789	0.037	0.13	.783
Foreign born	Yes	0.09	0.09	.296	0.115	0.13	.372

All analyses adjusted for time (days since start of treatment), using cubic splines with four knots.

Two‐tailed *p*‐value significance level .05.

a Severe malaria according to WHO criteria (TMIH 2014).

### 2.2 Telomerase expression dynamics

**Figure 2:**
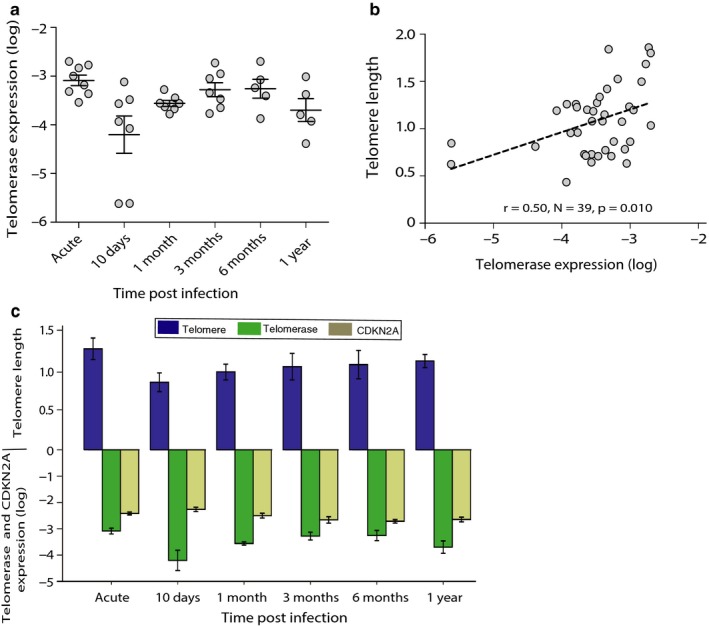
Relationship between telomere length and telomerase expression in blood of subset of travelers with RNA samples (*n *= 8, observations = 39) followed over one year after a successfully treated *Plasmodium falciparum* malaria infection. (a) Dynamics of telomerase expression level (log) after single acute malaria infection in peripheral blood; each gray circle represents the telomerase expression of individual sample, solid black lines represent the mean at each sampling point, and error bars denote the ± SE. (b) Pearson correlation (adjusted for cluster‐robust standard errors) between telomerase expression and telomere length. (c) Telomere length, telomerase expression level (log), and CDKN2A level (log) at the respective sampling time points. Bars represent the mean values and error bars denote the ± SE

Telomerase (hTERT) expression tended to be lower at 10 days postinfection compared to day 0 (paired t test, mean diff = −2.64, 95% CI 0.03 to −5.31, *N *= 7, *p *= .052) and was significantly lower at 1 month postinfection (mean diff = −1.06, 95% CI −0.31 to −1.81, *N *= 7, *p *= .013) compared to day 0, while telomerase expression was significantly higher at 3 months as compared with 10 days postinfection (mean diff = 2.72, 95% CI 5.01 to 0.44, *N *= 6, *p *= .028,) (Figure 2a, Table S2). Pearson correlation (adjusted for cluster standard errors) revealed a significant positive correlation between telomerase expression and telomere length (*r *= .50, *N *= 39, *p *= .010) (Figure 2b). The mixed effects models, with time modeled as a restricted cubic spline, showed that telomerase expression significantly predicted telomere length at any sampling time point (*N *= 39, *p *= .002) (Figure 2c), with no effect of age, sex, and patient origin (all *p *≥ .857, Table 2). Although telomerase activity predicted telomere length at the sampling time point, there was no significant correlation between telomerase expression and telomere length at the subsequent time point (*p *= .921).

**Table 2:** Telomere length association with telomerase expression and CDKN2A expression in blood of travelers (adjusted for other host factors) over one‐year follow‐up after a single successfully treated *Plasmodium falciparum* malaria infection (*N *= 8, observations = 39)

Factors	Coef.	SE	*p*‐value
Telomerase	0.09	0.03	**.002**
CDKN2A	−0.05	0.09	.584
Age	0.01	0.06	.861
Sex	−0.08	0.43	.857
Foreign born	0.10	0.72	.892

All analyses adjusted for time (days since start of treatment), using cubic splines with four knots.

Two‐tailed *p*‐value significance level .05. Significant *p*‐value are shown in bold.

### 2.3 CDKN2A expression dynamics

**Figure 3:**
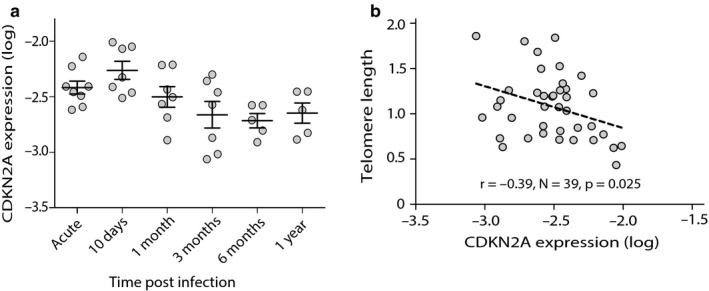
Relationship between telomere length and telomerase expression in blood of subset of travelers with RNA samples (*n *= 8, observations = 39) followed over one year after a successfully treated *Plasmodium falciparum* malaria infection. (a) Dynamics of CDKN2A level (log) after single acute malaria infection in peripheral blood; each gray circle represents the CDKN2A expression of individual sample, solid black lines represent the mean at each sampling point, and error bars denote the ± SE. (b) Pearson correlation (adjusted for cluster‐robust standard errors) between CDKN2A and telomere length

CDKN2A expression was significantly higher on day 10 postinfection compared to day 0 (mean diff = 0.33, 95% CI 0.65 to 0.01, *N *= 7, *p *= .045), and significantly lower at 3 months compared to day 10 postinfection (mean diff = −1.12, 95% CI −0.14 to −2.35, *N *= 6, *p *= .034) (Figure 3a, Table S3). Pearson correlation (adjusted for cluster standard errors) revealed a significant negative correlation between CDKN2A expression and telomere length (*r *= −.39, *N *= 39, *p *= .025) (Figure 3b), and there was a suggestion of a negative correlation between CDKN2A expression and telomerase activity (*r *= −.30, *N *= 39, *p *= .062) (Fig. S2). The mixed effects models, with time modeled as a restricted cubic spline, showed that CDKN2A expression did not significantly correlate with telomere length neither at sampling time point (*p *= .584) nor at the subsequent time point (*p *= .345), when controlling for age, sex and patient origin (Figure 2c, all *p *≥ .584 Table 2). Furthermore, CDKN2A expression and telomerase activity showed a tendency for a negative correlation at the respective sampling time points (*p *= .123) (Figure 2c, Table S4), when controlling for age, sex, and patient origin. However, there was no significant correlation of CDKN2A expression with telomerase activity at subsequent time point (*p *= .889) when controlling for age, sex and patient origin.

### 2.4 Differential cell count

We then analyzed whether the changes observed in peripheral blood telomere length were reflected by the variations in the proportion of different leukocyte phenotypes (including neutrophils, lymphocytes, and monocytes). The total leukocyte count did not significantly change over the follow‐up period (*p *= .501). However, the proportion of different leukocytes significantly changed over time (all *p *< .001), with an increase in lymphocytes and a decrease in monocytes and neutrophils after the acute malaria episode (Fig. S3). The mixed effects models, with time modeled as a restricted cubic spline, showed that the differential leukocyte counts were not significantly correlated with telomere length at the respective sampling time points over the one‐year‐long follow‐up (all *p *> .16, Table S5).

## 3 DISCUSSION

This is, to our knowledge, the first study that investigates the effect of malaria on aging in humans. Using a longitudinal approach, we find that a single malaria infection affects cellular aging; moreover that the effect was largely reversed after successful treatment and in the absence of new infections in individuals prospectively followed over a year.

Our results show that acute malaria infection leads to elevated levels of CDKN2A expression, lower telomerase activity, and substantial telomere shortening during the first three months postinfection. Although telomere shortening has been shown in acute infections in experimental animal models (Asghar, Hasselquist, et al., 2015; Asghar et al., 2016; Ilmonen et al., 2008), such effect has not been reported in humans. Previous studies have shown that chronic viral infections (i.e., HCV, CMV, and HIV) accelerate cellular aging in humans, as reflected by shorter telomere length and elevated CDKN2A expression (van de Berg et al., 2010; Gianesin et al., 2016; Leung et al., 2017; Pathai et al., 2013; Robinson et al., 2013; Zannetti et al., 2006). Furthermore, genome‐wide association studies (GWAS) have linked CDKN2A to many age‐related pathologies, including susceptibility to frailty and increased risk of coronary artery disease, myocardial infarction, type 2 diabetes, and Alzheimer's disease (Jeck et al., 2012). In addition, shorter telomere length and reduced expression of telomerase regulatory genes have been reported in experimental malaria studies in birds (Asghar et al., 2016; Videvall, Cornwallis, Palinauskas, Valkiunas, & Hellgren, 2015). Besides being a valuable cellular senescence marker, CDKN2A has also been suggested to regulate telomerase activity in stem cells, where overexpression of CDKN2A suppresses hTERT (which encodes the catalytic unit of telomerase) (Martin, Beach, & Gil, 2014). Our results also suggest a negative correlation between CDKN2A and telomerase (hTERT) expression. In the majority of human cell types, replicative senescence is primarily driven by progressive increase and/or overexpression of CDKN2A upon excessive cell division and other stressors (Sandhu, Peehl, & Slingerland, 2000). Extrapolating this to our results suggests that excessive replication of stem cells due to malaria infection may be responsible for the enhanced expression of CDKN2A, which suppresses telomerase expression and consequently telomere length shortens in blood cells.

In this study of acute malaria infection, we find distinct dynamics of cellular aging not observed during chronic infection (van de Berg et al., 2010; Gianesin et al., 2016; Leung et al., 2017; Pathai et al., 2013; Robinson et al., 2013; Zannetti et al., 2006). After successful treatment and in the absence of new infections, the effect on cellular aging markers was largely reversed as reflected by lower CDKN2A expression, higher telomerase activity, and gradual restoration of telomere length in peripheral blood of travelers from three months postinfection up to one year. Nonetheless, as we did not have access to blood samples before the diagnosis of malaria, we cannot know whether restoration of the cellular aging process is complete or only partial. The telomere length, telomerase, and CDKN2A expression at the time of diagnosis might have already been affected by the disease that had been ongoing for several days (median 4 days) before the travelers came under medical treatment. In the field of biomedical research, telomere length has often regarded as a biomarker for cumulative cellular damage. However, initial telomere length in peripheral blood might also affect the outcome of infection as demonstrated in a challenge study with respiratory viruses, where volunteers with shorter telomere length in blood cells showed higher susceptibility (Cohen et al., 2013).

We measured cellular aging markers (telomere length, CDKN2A, and telomerase expression) in whole blood. A limitation of our study is that we are not able to pinpoint whether malaria has a synchronized effect on cellular aging markers in all cell types or whether there are specific effects on certain cell subtypes. Nonetheless, telomere length measured in total peripheral blood mononuclear cells (PBMCs) has been shown to be highly correlated to different cell subtypes, and to be highly synchronized in monocytes, T cells, and B cells in healthy individuals (Lin et al., 2016). Furthermore, it has also been shown that there is a synchrony in telomere length and telomere loss in adult mammals and humans (Daniali et al., 2013). Here, we find that although cellular composition in malaria patients changes over one year, differential cell counts at the respective sampling time points did not correlate with telomere length. Notably, in an experimental malaria study of birds, telomere shortening was observed in whole blood as well as in other organs (liver, lungs, heart, spleen, kidney, and brain), suggesting that malaria infection causes systemic stress that results in parallel and synchronized telomere shortening in the whole body (Asghar et al., 2016). Future studies including cell sorting and single cell analyses are needed to investigate whether malaria has a synchronized effect on cellular aging markers in all cell types or whether there are specific effects on certain cell subtypes.

In malaria endemic areas, individuals are continuously exposed to infected mosquitoes and might face up to hundreds of infective bites per year. After repeated infections, individuals gradually acquire immunity that protects against disease but are unable to clear asymptomatic low‐level parasitemia (Doolan, Dobano, & Baird, 2009). Whether repeated malaria episodes or chronic asymptomatic infections have an even more pronounced and long‐lasting effect on cellular aging (and lifespan) in humans, as shown in birds with asymptomatic chronic malaria infection (Asghar, Hasselquist, et al., 2015), needs to be investigated. Such findings would have major implications on the need to further speed up the global efforts to eliminate malaria.

In summary, we show that a single acute *P. falciparum* malaria infection affects cellular aging dynamics in humans and that after successful malaria treatment, and in the absence of new malaria infection, the effect on cellular aging was largely reversed after one year. Hidden long‐term costs of malaria need to be established in populations living under repeated exposure in endemic areas. Moreover, these findings might not only be specific for malaria and cellular aging needs to be studied also in the context of other human infections. Such findings are important to elucidate the underlying cellular mechanism by which pathogens can affect host cellular aging and longevity.

## 4 METHODS

### 4.1 Prospective cohort sampling

The study was performed on adults diagnosed and treated for *P. falciparum* malaria (*n *= 38) at Karolinska University Hospital in Stockholm, Sweden. Written informed consent was obtained from all participants and the study was approved by the Ethical Review Board in Stockholm (Dnr 2006/893‐31/4 and 2013/550‐32/4). Venous blood was collected in EDTA at the time of malaria admission, and patients were then asked to return for blood sampling at 10 days, 1 month, 3 months, 6 months and 12 months after treatment. In a subset of patients (*n *= 8), blood was also collected in Tempus™ Blood RNA tubes for gene expression analysis. RNA collection was introduced later in the study; therefore, only a subset of patients has contributed with RNA samples.

The patients were aged between 26 and 65 years; 29 (74.3%) were male and 11 (29%) were born in Sweden. All patients were treated with six doses of artemether–lumefantrine (AL) (20 mg/120 mg Riamet^®^; four tablets per dose at 0, 8, 24, 36, 48 and 60 hr). Six patients received one to three initial dose(s) of intravenous artesunate (2.4 mg/kg per dose) before a full course of AL. The self‐reported duration of fever at time of diagnosis varied from 1 to 14 days (median 4 days; 1st – 3rd quartile: 3 – 5). Nine patients were categorized as severe malaria cases according to the WHO criteria (TMIH 2014), among them five patients had severe malaria using hyperparasitemia (>5% parasitemia) as a single criterion for severity.

### 4.2 Microscopy

Conventional light microscopy of Field's stained thin and thick smears was performed for species identification, detection, and enumeration of parasites (percentage infected erythrocytes) by expert microscopists at the Department of Clinical Microbiology, Karolinska University Hospital. Parasite densities ranged from < 0.1 to 21% infected erythrocytes corresponding to 500–1000,000 parasites per microliter blood (median 35000; 1st – 3rd quartile: 5000–97500).

### 4.3 Leukocyte differential count

Total and differential leukocyte counts were obtained using automated hematology analyzers, Sysmex XE5000 and Sysmex XN at the Department of Clinical Chemistry, Karolinska University Hospital, in accordance with the CLSI H20‐A2 standard. Samples flagged by automated hematology analyzers were counted manually.

### 4.4 Telomere length measurement

A magnetic bead separation method with Hamilton Chemagic Star Robot^®^ (Bonadouz, Switzerland) was used to extract DNA from 200 μl of blood. DNA was diluted to 1 ng/μl to quantify telomere length on QuantStudio 5 qPCR instrument, and qPCR runs were analyzed by Thermo Fisher Cloud Software. Relative quantitative PCR method using primers *tel1, tel2* to quantify telomere length and *HBG1, HBG2* was used to quantify a single‐copy gene to control for the total amount of DNA in each reaction (Cawthon, 2002). Each 25 μl reaction contained 5 μl (1 ng/μl DNA), 12.5 μl Supermix (Platinum SYBR Green qPCR Super Mix‐UDG, Invitrogen, CAT # 11733046, Thermo Fisher Scientific), 0.1 μl ROX, 0.3 μl (10 μmol/L) of each *tel* primer, or 1 μl (10 μmol/L) of each *HBG* primer and ddH_2_O. For telomere qPCR, the thermal cycle profile included incubation at 50°C for 2 min and 95°C for 10 min before running 30 thermal cycles (95°C for 15 s, 54°C for 45 s, and 72°C for 45 s). For single‐copy qPCR, the thermal cycle profile included incubation at 50 °C for 2 min and 95°C for 10 min before running 40 thermal cycles (95°C for 15 s, 58°C for 45 s, and 72 °C for 45 s).

Each 96‐well plate contained a reference sample (Gold control, to control interplate variability), two negative controls, and a serially diluted sample (25 ng, 12.5 ng, 6.25 ng, 3.12 ng, and 1.56 ng) to produce a standard curve. These serial dilutions of the standard were run on all plates for both sets of primers. All samples, reference, standards, and negative controls were run in duplicate for each primer set, and each primer set was run on different plates. The mean *C*_T_ values of duplicates were used in all calculations. From the *C*_T_ values of qPCRs, we calculated the amounts of the telomere sequence and the single‐copy nuclear sequence relative to their respective standard curves. These values were standardized across plates by dividing them by the plate value of the “golden sample” in order to obtain plate‐adjusted amounts of telomere sequence (T) and single‐copy nuclear sequence (S). Finally, we calculated the relative telomere length as the ratio of T and S (T/S). As the standard curves are expressed in ng / μl, this is the unit used to calculate T/S ratio; hence, this approach can easily be compared with ΔΔ*C*_T_ values as –log^2^ of T/S is equal to ΔΔ*C*_T_ (Asghar, Bensch, Tarka, Hansson, & Hasselquist, 2015; Cawthon, 2002). This T/S ratio was referred here as “telomere length.”

The within‐plate repeatability (i.e. between technical replicates of the same sample) for non‐normalized values was obtained by the primer sets *tel* and *HBG*. Six plates (three plates for each primer set *tel* and *HBG*) with each plate containing 25 samples were run in duplicate. Intraplate repeatability and interplate repeatability of telomere length measurements were analyzed using linear mixed effect models (LMM, R 2.15.1) fitted with restricted estimate maximum likelihood and including the sample id as a random factor. Intraplate repeatability was very high for both primers (*tel*, mean intraclass correlation (ICC)  = 0.98; *HBG*, mean ICC = 0.99). Our method also showed very high interplate repeatability (*tel*, mean ICC = 0.98; *HBG,* mean ICC = 0.99). Finally, we calculated the repeatability of the T/S ratio (i.e., our estimate of “telomere length” after normalization for both the “golden sample” and the total DNA content in the sample) using the same statistical method as above. T/S ratio also showed high repeatability (ICC = 0.98).

### 4.5 RNA Extraction and cDNA preparation

RNA was extracted from blood collected in Tempus™ Blood RNA tubes (CAT # 4342792; Applied Biosystem) using Stabilized Blood‐to‐CT™ Nucleic Acid Preparation Kit (Cat # 4449080, Ambion, Life Technology) according to the manufacturer′s instruction. After the RNA evaluation, approximately 200 μg RNA was converted to cDNA using the SuperScript^®^ VILO™ cDNA Synthesis Kit (CAT # 11754250, Invitrogen, Thermo Fisher Scientific) according to the manufacturer′s instructions.

### 4.6 Telomerase expression measurement

Relative telomerase expression was measured using the human TaqMan^®^ Copy Number Reference Assay, hTERT (CAT # Hs00972650_m1; Applied Biosystem) on a QuantStudio 5 qPCR instrument. The TaqMan^®^ GAPDH Assay (CAT # 4485712; Applied Biosystem) was used as an endogenous control. Each 15 μl reaction contained 3 μl cDNA, 7.5 μl TaqMan^®^ Multiplex Master Mix (CAT # 4461882; Applied Biosystem), 1 μl GAPDH Assay, and 1 μl TERT Essay and ddH_2_O. Thermal cycling condition was 95 °C for 20 s before running 45 thermal cycles (95°C for 01 s and 60°C for 20 s). Thermo Fisher Clouds Software was used to calculate *C*_T_ values. Telomerase expression was calculated as 2^−Δ*CT*^, where Δ*C*_T_ = *C*_T_ of target gene – *C*_T_ of control gene (GAPDH). The TERT/GAPDH ratio is referred to as “telomerase expression.”

### 4.7 CDKN2A measurement

CDKN2A was measured using the TaqMan^®^ Gene Expression Assay (CAT # HS00923894_m1; Applied Biosystem) on a QuantStudio 5 qPCR instrument. The TaqMan^®^ GAPDH Assay (CAT # 4485712; Applied Biosystem) was used as endogenous control and run in the same reaction to obtain more robust quantification. Each 15 μl reaction contained 3 μl cDNA, 7.5 μl TaqMan^®^ Multiplex Master Mix (CAT # 4461882; Applied Biosystem), 1 μl GAPDH Assay, and 1 μl CDKN2A Essay and ddH_2_O. Thermal cycling condition was 95 °C for 20 s before running 45 thermal cycles (95°C for 1 s and 60°C for 20 s). Thermo Fisher Clouds Software was used to calculate the *C*_T_ values. CDKN2A expression was calculated as 2^−Δ*CT*^, where Δ*C*_T_ = *C*_T_ of target gene – *C*_T_ of control gene (GAPDH). The CDKN2A/GAPDH ratio is referred to as “CDKN2A expression.”

### 4.8 Statistical analyses

Analyses were performed in Stata 13.1 or R 2.15.1 (lme4 package). Telomerase expression and CDKN2A expression were log‐transformed before statistical analyses. Paired t tests were performed for mean telomere length, telomerase expression, and CDKN2A expression at each measurement point to investigate the change from one time point to another over one‐year follow‐up time. Pearson correlation (adjusted for cluster‐robust standard errors) was used to investigate the relationship between telomerase expression and telomere length, CDKN2A expression and telomere length, and telomerase expression and CDKN2A expression.

Mixed model with restricted maximum likelihood (Harrell, 2001; Laird & Ware, 1982) was used to investigate the dynamics of telomere length in repeated samples over one year. Time in study was modeled using restricted cubic splines (Durrleman & Simon, 1989). A restricted cubic spline is a piecewise function, joined together by knots, and constrained to be linear before the first knot and after the last knots, otherwise taking the shape of cubic polynomials. Modeling time with restricted cubic splines is more flexible and makes fewer assumptions than modeling time as linear or log‐linear. All models involving splines were fit using four knots, placed as suggested by Harrell (Harrell, 2001). We adjusted for sex, age, parasite density, number of febrile days before treatment, and severity of disease. These adjustments were made both separately for each variable and jointly for all of them in mixed model. Mixed model with restricted maximum likelihood and time fitted as restricted cubic splines were performed to investigate the relationship between telomere length and telomerase expression; telomere length and CDKN2A expression; and CDKN2A expression and telomerase expression, respectively, at given time point or at subsequent time point controlling for age, sex, and patient origin.

Finally, dynamics of total leukocyte proportion as well as the counts of lymphocytes, neutrophils, and monocytes during follow‐up was investigated in relation to telomere length. Univariate mixed model with time fitted restricted cubic splines was used to investigate whether total cell count and differential cell proportions change over 12 months. Mixed model with restricted maximum likelihood and time fitted as restricted cubic splines were used to investigate whether total or differential cell proportion predicts telomere length at the time of sampling.

## CONFLICT OF INTEREST

The authors declare no conflicting interests.

## DATA AVAILABILITY

All data are available upon request to Muhammad Asghar (asghar.muhammad@ki.se) and Anna Färnert (anna.farnert@ki.se).

## AUTHOR CONTRIBUTIONS

MA, DH, and AF designed the study; MA, VY, MVH, and KS collected and processed the patient's samples; MA and MVH performed experiments; MA analyzed data, generated figures, and wrote the manuscript; UH and MA performed the statistical analysis; and MA and AF supervised the study. All authors have read and revised the manuscript.
