# Supplementary material for: Cellular aging dynamics after acute malaria infection: A 12‐month longitudinal study
Source: Aging Cell. 2017 Nov 16;17(1):e12702. doi: 10.1111/acel.12702 (PMC5771395; doi:10.1111/acel.12702)
Supplement: Supplementary file 1 [file ACEL-17-na-s001.pdf]

Supplementary Materials

Cellular aging dynamics after acute malaria infection: A 12-months longitudinal study

Muhammad Asghar[1]*, Victor Yman[1], Manijeh Vafa-Homann[1], Klara Sondén[1,2], Ulf Hammar[3], Dennis Hasselquist[4], Anna Färnert[1,2]

Supplementary Figures

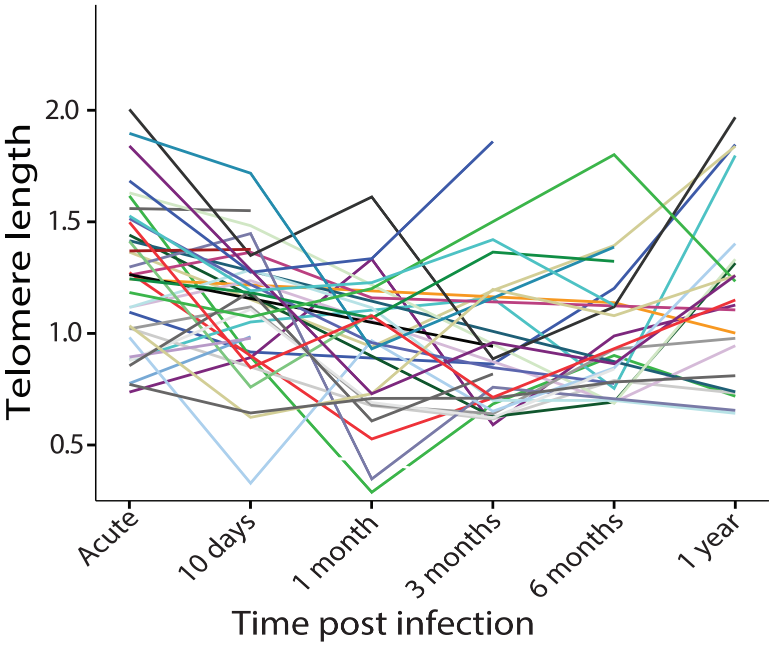

Fig. S1

Telomere length (T/S ratio) dynamics in blood of travelers followed one year after a successfully treated *P. falciparum* malaria infection. Each line represents an individual patient's telomere dynamics.

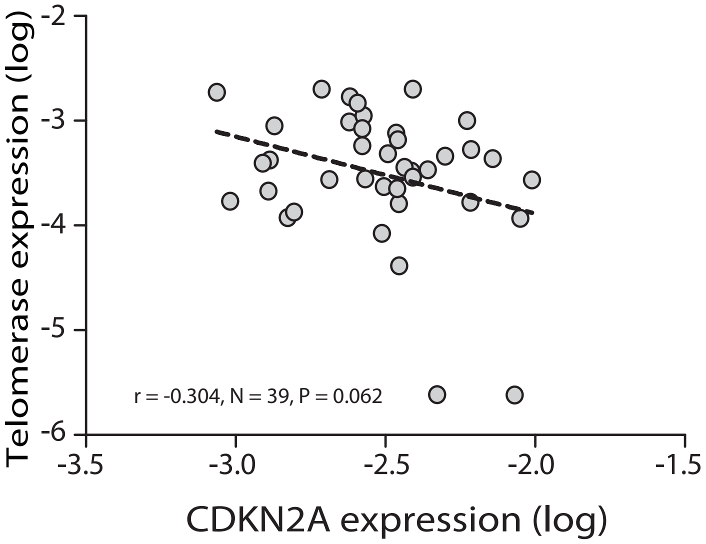

Fig. S2

Relationship between CDKN2A and telomerase expression (Pearson correlation, adjusted for cluster robust Standard Errors) in blood of subset of travelers (n=8) with RNA samples followed over one year after a successfully treated *P. falciparum* malaria infection.

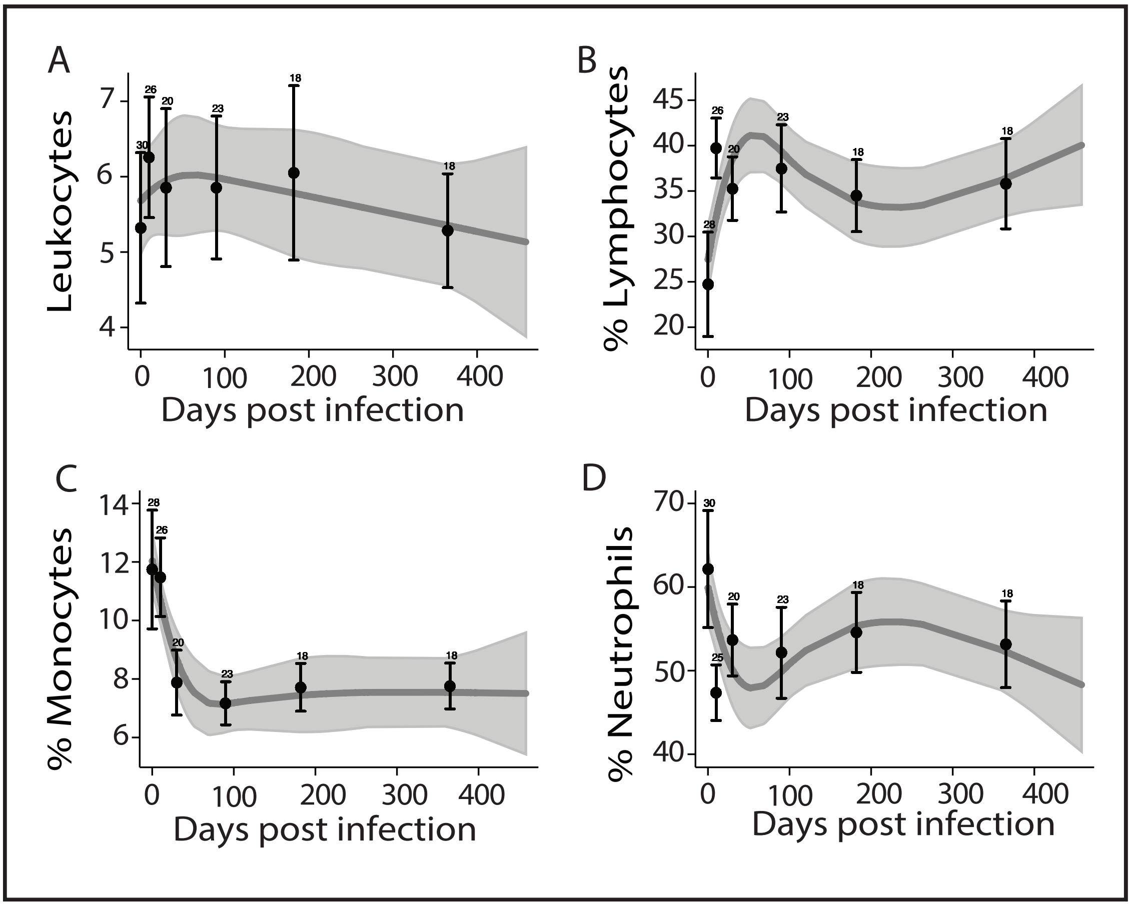

Fig. S3

Total leukocyte count (assuming $5x10^6$ erythrocytes per microliter whole blood) and proportion of different cell types in blood (%) over a one year follow up study in human travelers with successfully treated single malaria infection: (A) Leukocytes, (B) Lymphocytes, (C) Monocytes and (D) Neutrophils. Solid black lines denote the mixed model predicted mean proportion of cells (at the actual measurement time points) and the shaded gray areas denote the 95% CI of the model prediction. Black points denote the observed mean proportion of cells at each time point (pooled data at expected measurement day) and error bars denote the 95% CI of the mean. Numbers on each error bar represents the sample size. Lymphocytes, monocytes and neutrophils are presented as the % proportion of total leukocytes.

Supplementary Tables

Table S1: Paired t-tests predicting telomere length dynamics in blood in travelers over a year follow up. Mean difference of telomere length levels are presented as (Mean diff) and 95% confidence interval of mean difference as (95% CI).

	Day 10	*1 month*	*3 months*	*6 months*	*12 months*
**Acute (0)**	Mean diff = -0.14<br>95% CI = -0.02 - -0.25<br>N = 30**, p = 0.022**	Mean diff = -0.36<br>95% CI = -0.19 - -0.25<br>N = 25, **p < 0.001**	Mean diff = -0.34<br>95% CI = -0.19 - -0.48<br>N = 26, **p < 0.001**	Mean diff = -0.26<br>95% CI = -0.09 - -0.43<br>N = 24, **p = 0.004**	Mean diff = -0.07<br>95% CI = 0.12 - -0.26<br>N = 23, p = 0.460
**Day 10**		Mean diff = -0.11<br>95% CI = 0.07 - -0.31<br>N = 21, p = 0.233	Mean diff = -0.10<br>95% CI = 0.07 - -0.27<br>N = 20, p = 0.243	Mean diff = -0.07<br>95% CI = 0.11 - -0.26<br>N = 19, p = 0.411	Mean diff = 0.14<br>95% CI = 0.41 - -0.12<br>N = 17, p = 0.277
**1 months**			Mean diff = 0.10<br>95% CI = 0.17 - -0.15<br>N = 21, p = 0.902	Mean diff = 0.09<br>95% CI = 0.26 - -0.07<br>N = 18, p = 0.236	Mean diff = 0.21<br>95% CI = 0.38 - 0.03<br>N = 16, **p = 0.022**
**3months**				Mean diff = 0.07<br>95% CI = 0.17 - -0.25<br>N = 19, p = 0.134	Mean diff = 0.39<br>95% CI = 0.58 - 0.21<br>N = 17, **p < 0.001**
**6 months**					Mean diff = 0.26<br>95% CI = 0.45 - 0.06<br>N = 18, **p = 0.011**

Two tailed p-value significance level 0.05

Table S2: Paired t-tests predicting telomerase expression dynamics in blood in travelers over a year follow up. Mean difference of telomerase expression levels are presented as (Mean diff) and 95% confidence interval of mean difference as (95% CI).

	Day 10	*1 month*	*3 months*	*6 months*	*12 months*
**Acute (0)**	Mean diff = -2.64<br>95% CI = 0.03 - -5.31<br>N = 7, p = 0.052[a]<br>**p = 0.042[b]**	Mean diff = -1.06<br>95% CI = -0.31 - -1.81<br>N = 7, **p = 0.013**[a]<br>**p = 0.028[b]**	Mean diff = -0.59<br>95% CI = 0.03 - -1.21<br>N = 7, p = 0.059[a]<br>**p = 0.028**[b]	Mean diff = -0.09<br>95% CI = 1.81 - -1.98<br>N = 7, p = 0.902[a]<br>p = 0.892[b]	Mean diff = -1.26<br>95% CI = 0.67 - -3.21<br>N = 5, p = 0.145[a]<br>p = 0.138[b]
**Day 10**		Mean diff = 1.62<br>95% CI = 4.25 - -1.00<br>N = 6, p = 0.173[a]<br>p = 0.173[b]	Mean diff = 2.72<br>95% CI = 5.01 - 0.44<br>N = 6, **p = 0.028**[a]<br>**p = 0.027[b]**	Mean diff = 1.91<br>95% CI = 5.32 - -1.49<br>N= 4, p = 0.171[a]<br>p = 0.067[b]	Mean diff = 1.185<br>95% CI = 7.02 - -3.31<br>N = 4, p = 0.336[a]<br>p = 0.273[b]
**1 months**			Mean diff = 0.53<br>95% CI = 1.51 - -0.44<br>N = 6, p = 0.219[a]<br>p = 0.345[b]	Mean diff = 0.175<br>95% CI = 1.85 - -0.35<br>N = 5, p = 0.131[a]<br>p = 0.079[b]	Mean diff = -0.36<br>95% CI = 1.47 - -2.21<br>N = 5, p = 0.609[a]<br>p = 0.685[b]
**3months**				Mean diff = -0.37<br>95% CI = 1.99 - -2.07<br>N = 4, p = 0.957[a]<br>p = 0.715[b]	Mean diff = -0.94<br>95% CI = 1.36 - -3.25<br>N = 4, p = 0.283[a]<br>p = 0.273[b]
**6 months**					Mean diff = -1.55<br>95% CI = 0.43 - -3.15<br>N = 4, p = 0.054[a]<br>p = 0.068[b]

Paired t-test[a], Wilcoxon signed-rank test[b]
Two tailed p-value significance level 0.05

Table S3: Paired t-tests predicting CDKN2A expression dynamics in blood in travelers over a year follow up. Mean difference of CDKN2A levels are presented as (Mean diff) and 95% confidence interval of mean difference as (95% CI).

	Day 10	*1 month*	*3 months*	*6 months*	*12 months*
**Acute (0)**	Mean diff = 0.33<br>95% CI = 0.65 - 0.01<br>N = 7, **p = 0.045**[a]<br>p = 0.063[b]	Mean diff = -0.13<br>95% CI = 0.36 - -0.62<br>N = 7, p =0.539[a]<br>p = 0.499[b]	Mean diff = -0.89<br>95% CI = -0.16 - -1.61<br>N = 7, **p = 0.023**[a]<br>**p = 0.028**[b]	Mean diff = -0.77<br>95% CI = -0.32 - -1.22<br>N= 5, **p = 0.009**[a]<br>**p = 0.043**[b]	Mean diff = -0.55<br>95% CI = -0.06 - -1.04<br>N = 5, **p = 0.035**[a]<br>p = 0.079[b]
**Day 10**		Mean diff = -0.32<br>95% CI = 0.41 - -1.04<br>N = 6, p = 0.312[a]<br>p = 0.248[b]	Mean diff = -1.12<br>95% CI = -0.14 - -2.35<br>N = 6, **p = 0.034**[a]<br>**p = 0.046**[b]	Mean diff = -1.12<br>95% CI = -0.11 - -2.13<br>N = 4, **p = 0.038**[a]<br>p = 0.068[b]	Mean diff = -1.10<br>95% CI = -0.18 - -2.02<br>N = 4, **p = 0.031**[a]<br>p = 0.067[b]
**1 months**			Mean diff = -0.67<br>95% CI = 0.25 - -1.60<br>N = 6, p = 0.11[a]<br>p = 0.074[b]	Mean diff = -0.50<br>95% CI = 0.45 - -1.44<br>N = 5, p = 0.224[a]<br>p = 0.224[b]	Mean diff = -0.17<br>95% CI = 0.67 - -1.02<br>N = 5, p = 0.601[a]<br>p = 0.500[b]
**3months**				Mean diff = -0.35<br>95% CI = 1.16 - -1.87<br>N = 4, p = 0.508[a]<br>p = 0.465[b]	Mean diff = 0.45<br>95% CI = 2.04 - -1.13<br>N = 4, p = 0.430[a]<br>p = 0.465[b]
**6 months**					Mean diff = 0.24<br>95% CI = 0.31 - 0.16<br>N = 4, **p = 0.002**[a]<br>p = 0.067[b]

Paired t-test[a], *Wilcoxon signed-rank test*[b]
Two tailed p-value significance level 0.05

Table S4: Relationship between telomerase expression and CDKN2A in multivariate mixed effects models, with time modeled as a restricted cubic spline (N = 39, patients = 8).

Factors	*Coef.*	*SE.*	*p-value*
Telomerase	-0.10	0.06	0.123
Age	0.02	0.09	0.378
Sex	-0.07	0.65	0.912
Foreign born	-0.21	1.09	0.846

All analyses adjust for time (days since start of treatment), using cubic splines with 4 knots.
Two tailed p-value significance level 0.05

Table S5: Relationship between differential proportion of leukocytes with telomere length in multivariate mixed effects models, with time modeled as a restricted cubic spline (N = 135, patients = 34).

Factors	*Coef.*	*SE.*	*p-value*
Leukocytes	-0.02	0.01	0.160
Lymphocytes	0.001	0.001	0.747
Monocytes	0.01	0.01	0.166
Neutrophils	-0.001	0.001	0.303

All analyses adjust for time (days since start of treatment), using cubic splines with 4 knots.
Two tailed p-value significance level 0.05
